# Mesenchymal stem cells paracrine proteins from three‐dimensional dynamic culture system promoted wound healing in third‐degree burn models

**DOI:** 10.1002/btm2.10569

**Published:** 2023-07-02

**Authors:** Yingwei Wang, Jiaxin Wu, Jiamin Chen, Cheng Lu, Jinchao Liang, Yingyi Shan, Jie Liu, Qi Li, Liang Miao, Mu He, Xiaoying Wang, Jianhua Zhang, Zheng Wu

**Affiliations:** ^1^ Department of Ophthalmology The First Affiliated Hospital of Jinan University Guangzhou China; ^2^ Key Laboratory for Regenerative Medicine, Ministry of Education, Department of Developmental and Regenerative Biology Jinan University Guangzhou China; ^3^ Burn plastic surgery Longgang Central Hospital Shenzhen China; ^4^ Department of Biomedical Engineering Jinan University Guangzhou China; ^5^ Special Wards The First Affiliated Hospital of Jinan University Guangzhou China

**Keywords:** 3D‐dynamic culture system, mesenchymal stem cell, paracrine protein, polyethylene glycol temperature‐sensitive hydrogel, scar inhibition, skin regeneration

## Abstract

Recovery of skin function remains a significant clinical challenge for deep burns owing to the severe scar formation and poor appendage regeneration, and stem cell therapy has shown great potential for injured tissue regeneration. Here, a cell‐free therapy system for deep burn skin was explored using mesenchymal stem cell paracrine proteins (MSC‐PP) and polyethylene glycol (PEG) temperature‐sensitive hydrogels. A three‐dimensional (3D) dynamic culture system for MSCs' large‐scale expansion was established using a porous gelatin microcarrier crosslinked with hyaluronic acid (PGM‐HA), and the purified MSC‐PP from culture supernatant was characterized by mass spectrometric analysis. The results showed the 3D dynamic culture system regulated MSCs cell cycle, reduced apoptosis, and decreased lactic acid content, and the MSC‐PP produced in 3D group can promote cell proliferation, migration, and adhesion. The MSC‐PP + PEG system maintained stable release in 28 days of observation in vitro. The in vivo therapeutic efficacy was investigated in the rabbit's third‐degree burn model, and saline, PEG, MSC‐PP, and MSC‐PP + PEG treatments groups were set. The in vivo results showed that the MSC‐PP + PEG group significantly improved wound healing, inhibited scar formation, and facilitated skin appendage regeneration. In conclusion, the MSC‐PP + PEG sustained‐release system provides a potentially effective treatment for deep burn skin healing.

## 1 INTRODUCTION

Burn remains one of the most common injuries worldwide causing over 265,000 death every year.^1^ Third‐degree burn refers to the burn of the full‐thickness skin, including epidermis deep dermis and total degeneration and necrosis of the skin appendages, and even reach the subcutaneous, muscular, and skeletal levels.^2^ In clinical practice, large area third‐degree burn, total body surface area (TBSA) greater than 30%, treatments usually require skin‐grafting, while small area third‐degree burn (less than 10% TBSA) is more commonly seen and could be self‐repairing.^3^, ^4^ But still, varying degrees of scarring and loss of appendages after third‐degree burn wound healing could cause severe contracture, painful itching, and loss of skin function.^5^ Therefore, minimizing wound scarring and recovery of skin appendages are important issues for the treatment of third‐degree burn.

Mesenchymal stem cells (MSCs) have shown a great therapeutic potential to promote wound re‐epithelialization, reduce scars restore, and facilitate skin appendages regeneration.^6^ Many studies supported that the paracrine effects of MSCs were vital mechanisms for skin regeneration by regulating the inflammatory response, improving host cell recruitment, facilitating angiogenesis, and accelerating re‐epithelialization.^7^, ^8^, ^9^ Despite the effectiveness of direct cell transplantation, limitations such as the fragile cell activity in vitro, the harsh environment existing in vivo, and the unstandardized cell product could lower the therapeutic effect of transplanted cells for skin regeneration in the clinic.^10^, ^11^ As mesenchymal stem cell paracrine proteins (MSC‐PP) has similar therapeutic effects to that of MSC while avoiding the drawbacks of cell transplantation therapy, exploring MSC‐PP as a cell‐free therapy treatment for third‐degree burn regeneration could be a feasible strategy.

The secretion of MSC‐PP is greatly related to stem cell bioactivities and they are in vitro cultural environment. To obtain more suitable proteins for regenerative therapy as well as to increase the MSC‐PP secretion levels, many researchers have developed various cell pretreatment methods, such as hypoxia pretreatment, serum‐free culture, and inflammatory factor pretreatment, and favorable outcomes were achieved.^12^, ^13^, ^14^ Besides the above progresses, three‐dimensional (3D) culture has also been brought up as an effective culture strategy for large‐scale cell production as well as improving cell bioactivities.^15^ However, providing suitable physiological microenvironment for the cells remains a key issue. A previous study developed a dynamic perfusion system for the in vitro culture of high‐density multilayered MSC cell sheet, which could efficiently provide metabolic substrates for the cells and remove metabolic waste.^16^ On this basis, establishing an efficient 3D MSC dynamic culture system, and further investigating the secretion and protein function of MSC‐PP in the 3D system could be beneficial for exploring MSC‐PP as a cell‐free treatment option for skin burns.

Furthermore, the method of MSC‐PP administration is another key issue that would affect the effectiveness of injured skin regeneration. Following cell transplantation, the surviving transplanted cells could continuously secrete various proteins in the damaged area to achieve regenerative repair.^17^ On the contrary, direct injection of MSC‐PP may cause the loss of proteins in the damaged area, so it would be difficult to maintain an effective MSC‐PP dosage.^18^ Currently, using hydrogels as a drug delivery carrier has shown favorable efficacy for wound healing.^19^ Therefore, developing a hydrogel‐based delivery system for MSC‐PP mass release could be considered as a promising strategy for wound healing and long‐term skin regeneration.

To explore an efficient cell‐free therapy strategy for deep burn, the present study established a 3D dynamic culture system to obtain MSC‐PP as well as analyzing their specific function. A sustained‐release MSC‐PP system was established using a polyethylene glycol (PEG) thermosensitive hydrogel. Furthermore, the therapeutic effects of the MSC‐PP + PEG system on wound healing, scar prevention, and skin appendage regeneration were examined in the third‐degree burn rabbit model.

## 2 MATERIALS AND METHODS

### 2.1 Umbilical cord MSC isolation, culture, and characterization

Ethical approval was obtained from the Hospital Ethics Committee and the Ethics Committee of Jinan University (KY‐2020‐038). The umbilical cord was taken from consenting patients delivering full‐term infants by a caesarian or normal section from the First Affiliated Hospital of Jinan University. The umbilical cords were rinsed several times with PBS buffer. The arteries and veins were removed, and the remaining Wharton jelly was cut into cubes of approximately 1 cm^3^ and placed in flask culture dishes. The culture medium contained DMEM (Corning), supplemented with 10% fetal bovine serum (FBS, Corning), 1% glutamine (Gibco), and 1% penicillin–streptomycin (Gibco). After cells migrated from the jelly tissue to the dishes, the culture medium was replaced every 2 days.

2 × 10^4^ MSCs were seeded onto cell slides (Sorfa, 200300) and cultured for 24 h. Cells were incubated with the corresponding primary antibodies at 4°C overnight as follows: CD29 (Abcam, ab179471), CD90 (Abcam, ab181469). To follow, corresponding secondary antibodies, a goat anti‐mouse 488 secondary antibody (1:200, Invitrogen) and a goat anti‐rabbit 594 secondary antibody (1:200, Invitrogen) were added and incubated at 37°C for 2 h. The nuclei were counterstained with Hoechst 33528 (1:500, Invitrogen). Results were observed and recorded by the laser confocal scanning microscope (LSM 880 with Airyscan, ZEISS). The MSC surface markers identification CD29 (Abcam, ab179471), CD90 (Abcam, ab181469), CD45 (Proteintech, 20103‐1‐AP) were examined by flow cytometry (*n* = 3).

### 2.2 Modification and characterization of PGM‐HA

Porous gelatin microcarrier (PGM, Sigma) was activated in an EDC (Sigma)/NHS (Sigma) solution for 15 min in 4°C, and EDC/NHS solution was washed off in a 1000D dialysis bag filled with pure water. The PGM was immersed in a solution containing 400 μg/mL HA(Sigma) and EDC/NHC under ultrasonic shaking (100 w, 37 HZ) for 4 h, and then incubated in a shaker at room temperature for 48 h. Sequentially, the PGM should be placed in an ice box at −80°C for 12 h and freeze‐dried, and the PGM‐HA was prepared. Immunofluorescence, Fourier‐transform infrared spectroscopy (FTIR), and water absorption rate detection were performed to analyze the characteristic of PGM‐HA (*n* = 3). PGM and PGM‐HA were weighed by an analytical balance to get the dry weight as m_0_. PGM‐HA and PGM were frozen at −30°C overnight and lyophilized for 2 days. The dry weights of PGM‐HA and PGM were recorded respectively, then the two groups were placed at 37°C, and their weights were measured at 1, 5,10, 20, and 30 min as m_t_. The water absorption rate of the samples was calculated using Equation (1).

(1) $$\text{Water absorption rate}=\frac{\left(m_{t}-m_{0}\right)}{m_{t}}\times 100\%$$

Cell viability was examined to test the cytotoxic effect of PGM‐HA (*n* = 5). 2000 MSCs were seeded in 96‐well plates per well, and 100 μL culture medium with 24 h PGM‐HA extract was added to each well, and PGM extract and fresh culture medium were taken as control groups. 10 μL CCK8 (Dojindo, CK04) was added and incubated for 2 h every day for five consecutive days, and the optical density (O.D) values were recorded at 450 nm wavelengths for each well, and the cell proliferation rate in each group was calculated according to the O.D values of day 1.

### 2.3 Establishment of the 3D dynamic system

After MSC reached a confluency of 90% in the culture dish, the adherent cells were harvested with 0.25% trypsin‐ethylene diamine tetra acetic acid (EDTA, Gibco). 0.8 g dried weight PGM‐HA was used for each 3D dynamic culture system. P3 MSC was mixed with PGM‐HA at a density of 5000 cells/cm^2^ in a 50 mL centrifuge tube, and then MSC‐PGM‐HA was transferred to a 500 mL spinner flask and connected to a dynamic perfusion system described in the previous study,^23^ which consist of a peristaltic pump (IPC‐N Ismatec, Glattbruch‐Zurich) and O_2_ exchange equipment (Minucells, Regensburg). Following the assembly of the 3D dynamic culture system, the MSCs were allowed to adhere on the PGM‐HA under intermittent stirring at 30 rpm for 2 min every 30 min for 32 cycles (16 h) with 150 mL culture medium. For cell expansion process, the stirring regime was set as continuous stirring at 30 rpm and the total culture medium volume was 300 mL. DNA, protein, and glucose of the 3D culture system were tested by DNA extraction kit (Purelink), BCA protein quantification kit (CWBIO, CE0014S), and Glucose Content Assay Kit (Solarbio). All the performances followed the experimental procedures provided on the kit.

### 2.4 Cell bioactivity in the 2D and 3D culture Systems

MSC was cultured until day 9, and then the cells and culture medium in the dynamic system were collected as samples for a subsequent test. MSC‐PGM‐HA was freeze‐dried by CO_2_ supercritical extraction, then the dried samples were gold‐coated with platinum and the samples were observed and photographed by a scanning electronic microscope (SEM, Ultra 55, ZEISS). Meanwhile, MSC‐PGM‐HA was seeded on a monolayer dish to observe its morpho gram. Cells culturing in the 2D and 3D culture systems were harvested using trypsin. Cell cycle, apoptosis, and ATP concentration were measured utilizing PI stain kit (Leagene), Annexin V‐FITC/PI Apoptosis Kit (Elabscience), and ATP cartridge (Promrga), following manufacturer instructions(*n* = 3). The conditioned medium of the 3D group and the 2D group was used as the sample for cellular lactic acid content detection. Following the procedure provided in the lactic acid kit (Nanjing Jiancheng), a spectrophotometer was used to measure the absorbance of the sample at 530 nm.

### 2.5 Preparation of MSC‐PP

For MSC‐PP preparation, serum free supernatant conditioned medium was collected. Briefly, when the MSCs was cultured in the 3D system until day 9, and the cells were washed with PBS for twice for completely removal of FBS, the complete culture medium would be replaced with serum free culture medium (DMEM supplemented with, 1% glutamine and 1% penicillin–streptomycin). The serum free condition medium was collected following 48 h culture, and the collected serum free conditioned medium was added into the dialysis bag (MD77‐5 M, MwCo: 1000D) and put in the saline for dialysis. Saline was replaced every 4 h. The conductivity of saline following dialysis was examined, and dialysis would be stopped when the tested conductivity was equal to normal saline. The concentrated condition medium samples were filtered through a 0.22‐μm filter mesh. The protein concentration of all samples was measured by the bicinchoninic acid (BCA) Protein Assay Kit (Cwbio).

### 2.6 Mass spectrometric analysis of MSC‐PP

The sample preparation followed the procedure as reported previously.^20^ Data‐dependent acquisition (DDA) mass spectrum techniques were used to acquire tandem MS data on a ThermoFisher Q Exactive mass spectrometer (ThermoFisher, USA) fitted with a Nano Flex ion source. Data were acquired using an ion spray voltage of 1.9 kV, and an interface heater temperature of 275°C. For a full mass spectrometry survey scan, the target value was 3 × 10^6^ and the scan ranged from 350 to 2000 m/z at a resolution of 70,000 and a maximum injection time of 100 ms. For the MS2 scan, only spectra with a charge state of 2–5 were selected for fragmentation by higher‐energy collision dissociation with a normalized collision energy of 28. After collecting mass spectrometry data, Gene Ontology (GO) and Kyoto Encyclopedia of Genes and Genomes (KEGG) enrichment analyses were conducted (*n* = 3).

### 2.7 Preparation of PEG thermogel

Purified dried PEG (M_n_ = 1500, Shanghai Aladdin Bio‐Chem Technology Co), Sn(Oct)_2_(Aldrich) of the toluene solution, *ɛ*‐CL(Acros) and 1,4,8‐trioxaspiro[4.6]‐9‐undecanone (TOSUO) were added to ampoules, stirred while vacuuming, and the moisture in the system was removed by the azeotropic effect of toluene.^21^ The melt sealing tube was carried out by using an alcohol blowtorch, placed in an oil bath pot, and adjusted to 120°C for 16 h, and the PEG copolymer was obtained. An appropriate amount of copolymer (20%) was weighed and added to acetone until it was completely dissolved, using a syringe pump to slowly inject it into distilled water, and stirring to make acetone completely volatilized in a high speed, stored at 4°C for 12 h.

### 2.8 Characterization of PEG thermogel

The solution‐gel (Sol–gel) phase transition diagram of the PEG micelle solution was determined by the vial reversal method. Polymer aqueous solutions were prepared by the solvent‐exchange method described previously and then 1 mL of the solutions were transferred to 4 mL vials before being subject to heat treatment in a water bath starting at 15°C at a heating rate of 1°C/min. At each temperature point, the sol–gel transition was confirmed by a flow (sol)‐no flow (gel) criterion when the vial was inverted for 30 s.

The gelation temperatures, gelation time at 37°C as well as gel strength of the PEG gel were determined by a Fluids Rheometer (Malvem, Kinexus Pro). The polymer aqueous solution of 20% (w/t) was kept below 4°C before being placed between a 2° core plate with a diameter of 60 mm and a gap of 0.07 mm for temperature sweep and time sweep.

The cytotoxicity of PEG thermogel was examined using a CCK‐8 kit. The PEG thermogel was immersed in a complete medium at 37°C for 72 h at a ratio of 0.2 g/mL to obtain a hydrogel extract. Dermal fibroblasts were planted in 96‐well plates at a planting density of 1000 per well. The hydrogel extract and complete medium were separately added, and the absorbance after the reaction with CCK‐8 solution for 5 days was continuously measured. The absorbance of the first day of each group was used as a reference to calculate the change in dermal fibroblast activity.

### 2.9 Release behavior study of PEG thermogel loaded with MSC‐PP

PEG thermogel loaded with MSC‐PP was prepared by direct mixing PEG micelle solution with a specific concentration (30, 35, and 40 mg/mL). After solidification, the mixed micelle solution was taking a water bath at 37°C to form MSC‐PP‐released hydrogels. Each group was taken 1 mL to form a gel, and after 30 min, 10 mL PBS buffer solution was added to the tube and warm up to 37°C. Test samples were taken 4 mL release liquid every time, and 4 mL fresh PBS was replaced. Subsequently, BSA concentration in the released solution was detected by ultraviolet spectrophotometer, and the mass of BSA in the released solution was calculated according to the pre‐determined standard curve, and the cumulative release curve was calculated.

### 2.10 Migration capability of MSC‐PP on rabbit dermal fibroblasts and keratinocytes

4.0 × 10^5^ rabbit dermal fibroblasts were cultured in six‐well plates, and a straight linear wound was made by using a sterile 1000 μL pipette tip to scratch perpendicular to the cell layer. After that, the medium containing 0, 1, 1.5, and 2 mg/mL MSC‐PP were added respectively. The size of wounds was observed and measured at 0, 6, and 12 h after scratching. For keratinocytes examination, 5.0 × 10^5^ rabbit keratinocytes were cultured in 12‐well plates. When the cells reached 90% density, the scratching was carried out, and the medium containing 0 mg/mL, 1 mg/mL, 1.5 mg/mL, and 2 mg/mL MSC‐PP were added respectively. The size of wounds was observed and measured at 0 h, 12 h, and 24 h after scratching. The distance between both sides of the scratch was measured by Image Pro‐Plus software (Media Cybernetics), the distance at 0 h was marked as s_0,_ and distances at measure time points were marked as s_t_. The migration rate at different times was calculated using Equation (2):

(2) $$\text{Migration rate}=\frac{s_{0}-s_{t}}{\text{time}}\times 100\%$$

### 2.11 The effects of MSC‐PP on rabbit dermal fibroblasts in an inflammatory environment

10 ng/mL TNF‐α was added in the medium containing different content of MSC‐PP to culture dermal fibroblasts. CCK8 was used for cell viability assay as described above. The transformation of dermal fibroblasts into myofibroblasts in an inflammatory culture environment was detected by western blot. To be specific, cell proteins were extracted by using RIPA lysate and followed by the total concentration of proteins using a BCA kit. The SDS‐PAGE gel electrophoresis was then performed. The film was dissolved and closed at room temperature using BSA, and then the membrane was incubated overnight with β‐actin, α‐SMA, followed by HRP‐labeled sheep anti‐mouse immunoglobulin II anti‐incubation, and finally using the ECL chemical luminescent kit to detect antibody binding protein. Image J software was used to measure the densitometry of the immunoreactive bands. β‐actin was regarded as an internal control.

### 2.12 Animal models and therapeutic experiments

The animal experiment was conducted following the ethical guidelines of the National Guide for the Care and Use of Laboratory Animals and approved by Jinan University Animal Care and Use Committee (Approval numbers: IACUC‐20220722‐03). Third‐degree burn models were carried out in New Zealand rabbits (3‐month‐old, male, 1.8–2.5 kg, *n* = 17) by using a scald device. In brief, following anesthesia by ear vein injection of pentobarbital sodium (30 mg/kg, sigma). After anesthesia, the animals were placed on the squat plate to fix the limbs. The 1.7 cm^2^ metal block was installed on the temperature control burner. The temperature was adjusted to 100°C, the scald time was 15 s, the pressing force was 500 g, and four wounds were caused on the back of the animal.

Deep skin burns in rabbits were treated with the MSC‐PP + PEG sustained release system (the depth of the needle feed is 4–5 mm). A total of 17 experimental animals were set up in the Saline group, PEG group, MSC‐PP group, and MSC‐PP + PEG group. One wound was selected as the saline group on the back of each animal, and the other three wounds were used as the drug treatment group. To avoid interactions between each wound, the wounds would keep a distance above 5 cm. For the experimental groups, 0.5 mL MSC‐PP + PEG containing 40 mg/mL MSC‐PP was injected subcutaneously injected in the wound. In addition, 0.5 mL 3 mg/mL MSC‐PP was injected into the wound in MSC‐PP group every other day, and the total concentration of MSC‐PP injection over 4 weeks was consistent with the MSC‐PP + PEG group. Then 0.5 mL PEG hydrogel and 0.5 mL normal saline were injected subcutaneously into the wound, respectively. Finally, the wounds were wrapped with sterile cotton and gauze. For a total of 4 weeks of wound healing, three experimental animals were chosen at random at week 1, week 2, and week 3, and eight experimental animals were available at week 4. For wound area calculation, the wound area was measured by tracing the wound border on image J at the time points of week 1, week 2, week 3, and week 4. The “n” represents the week, like week 1, week 2, week 3, and week 4. The percentage of wound area (%) was calculated using Equation (3):

(3) $$\text{Wound area}\;\left(\%\right)=\frac{\text{wound area}\;\left(n\;\text{week}\right)}{\text{wound area}\;\left(0\;\text{week}\right)}\times 100\%$$

### 2.13 Histological analysis

Skin trauma tissue was fixed in 4% paraformaldehyde for 24 h, dehydrated in a series of graded ethanol, paraffin soaked, and cut into 5 μm thick sections. Sections were gradually hydrated with xylene and a series of graded ethanols. Hematoxylin–eosin (H&E) staining and Masson trichrome staining were then performed, and quantitative calculations were performed by ImageJ to assess wound re‐epithelialization, skin appendages, epidermal thickness, inflammatory cells, and vascular regeneration.

### 2.14 Immunofluorescence analysis

Sections were subjected to a heat‐mediated antigen repair step and incubated with the following primary antibodies: a mouse monoclonal antibody against type I collagen (1:200, Abcam), a rabbit monoclonal antibody against type III collagen (1:200, Abcam), Anti‐DLK‐1 antibody (1:150, Abcam), Anti‐CD26 antibody (1:100, Abcam), Anti Lgr6 antibody (1:200, Abcam), Anti‐Sca‐1antibody (1:200, Abcam), Anti β‐Catenin antibody (1:200, Abcam), Anti KRT5 antibody (1:400, Abcam), and incubated in appropriate secondary antibodies for 2 h. Hoechst 33258 (1:500, Invitrogen) was used for nuclear detection.

### 2.15 Statistical analysis

All analyses were performed using GraphPad Prism 9.0 (GraphPad Software Inc). For comparisons of two groups, a two‐tailed Student's *t* test was used. Comparisons of multiple groups were made using one‐ or two‐way ANOVA. The number of samples in each experimental group was greater than or equal to three (*n* ≥ 3). All quantitative results were expressed as the mean ± standard deviation, and differences with a *p* value<0.05 were considered statistically significant.

## 3 RESULTS

### 3.1 Establishment of a 3D dynamic culture system

**FIGURE 1:**
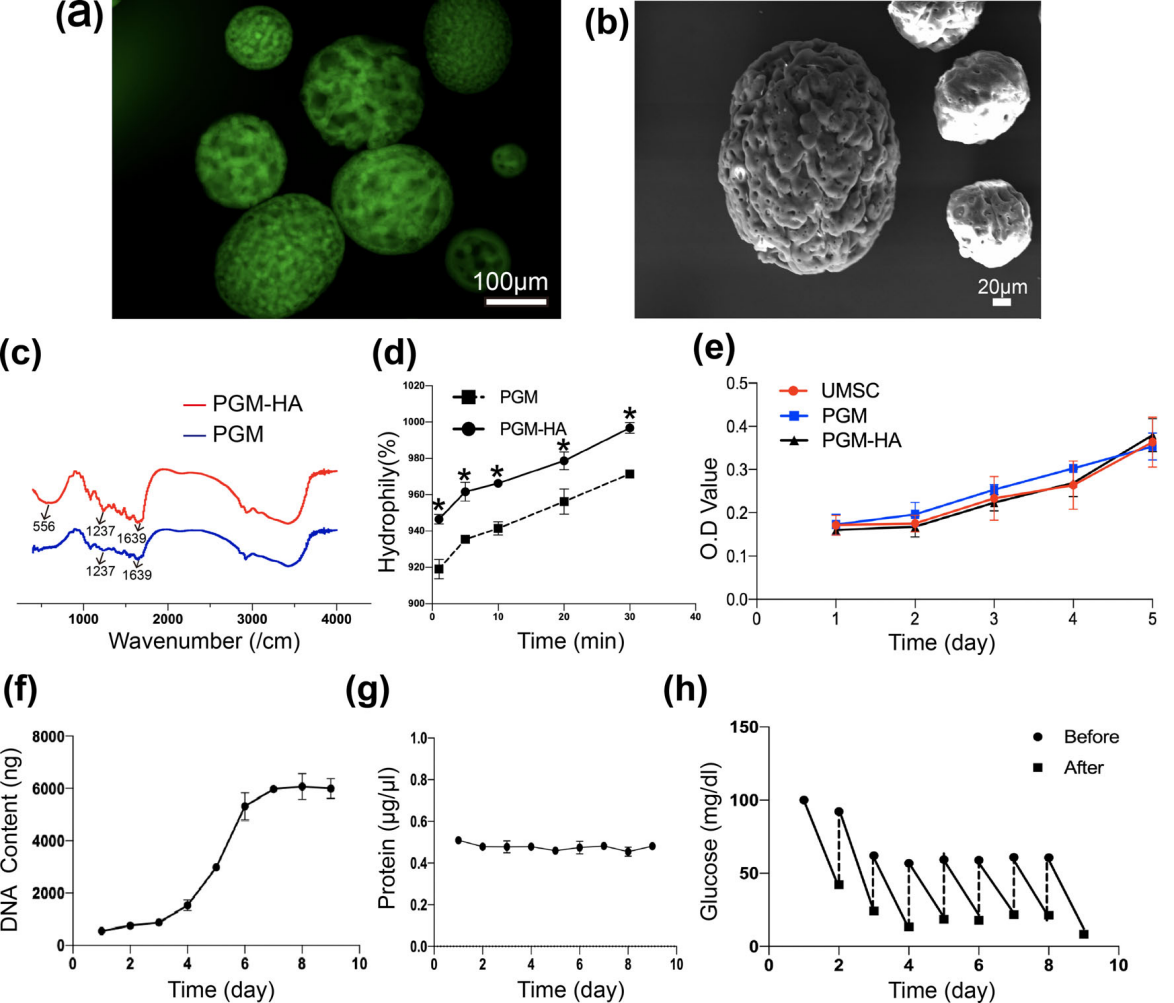
Preparation of a three‐dimensional dynamic culture system. (a) Immunofluorescence staining of PGM‐HA. (b) SEM result of PGM‐HA. (c) FITR results of PGM and PGM‐HA. (d) The water absorption rate of PGM and PGM‐HA. *p < 0.05 versus PGM. (e) PGM‐HA cell toxicology test over 5 days. (f) DNA content change chart over 9 days in the culture system. (g) Change the charge of protein in the culture medium during dynamic culture. (h) Glucose consumption of the culture system over 9 days (*n* = 3). All data are shown as the mean ± SD. PGM‐HA, porous gelatin microcarrier crosslinked with hyaluronic acid.

Labeling HA with green fluorescence and the fluorescence signal was observed on PGM in Figure 1a. SEM analysis demonstrated that the ultrastructure of PGM‐HA was porous (Figure 1b). The FITR spectroscopy result showed stronger bands at 1237 and 1639 cm^−1^ in PGM‐HA compared to PGM (Figure 1c). These changes indicated that HA was crosslinked with PGM by an esterification reaction. After lyophilizing PGM‐HA and PGM, the rehydration rate of PGM‐HA was significantly higher than that in the PGM group (Figure 1d). No significant difference in MSC proliferation was observed between the PGM‐HA extract culture medium and the fresh culture medium, indicating PGM‐HA was not cytotoxic (Figure 1e). After seeding MSC on PGM‐HA in the 3D dynamic culture system, the DNA content increased from day 1 to day 9, which indicated that the constructed 3D dynamic culture system could achieve 11‐fold cell expansion in 9 days (Figure 1f). The curve of protein concentration change was almost straight, and the protein concentration remained relatively stable from inoculation (day 0) to day 9 (Figure 1g). The glucose content in the medium was measured before and after the exchange. The results showed a decrease in glucose concentration in 9 days, which indicated the glucose concentration remained stable (Figure 1h).

### 3.2 Changes in the morphology and function of MSC under 3D dynamic culture

**FIGURE 2:**
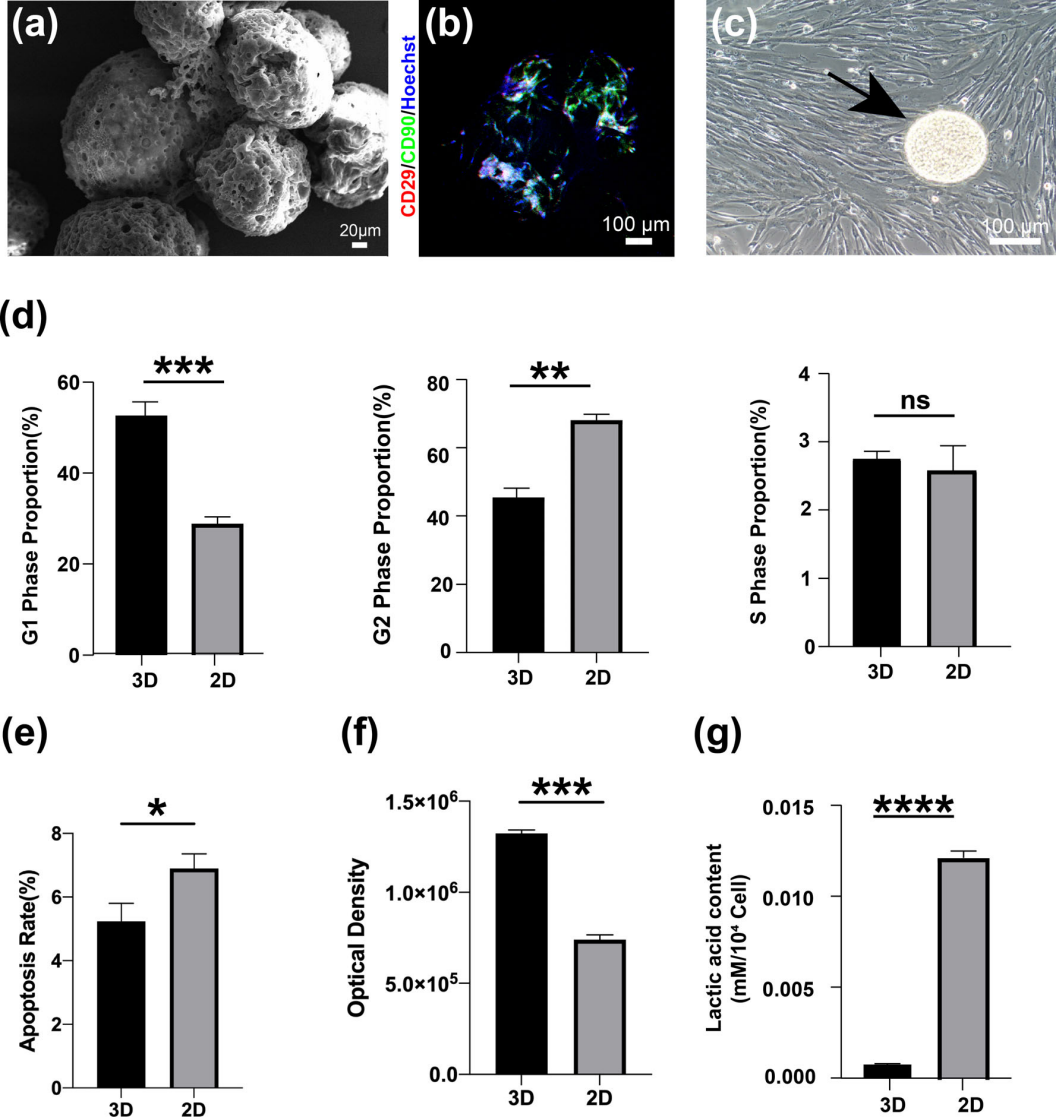
Changes in the morphology and function of MSC under 3D dynamic culture. (a) SEM result of MSC‐PGM‐HA. (b) Immunofluorescence staining result of CD90 (green) and CD29 (red) in MSC‐PGM‐HA. (c) Morphogram of MSC cultured on PGM‐HA after three‐dimensional dynamic culture. The black arrow indicates PGM‐HA. (d) Cell cycle analysis of MSC in the 3D group and the 2D group. (e) Cell apoptosis of MSC in the 3D group and the 2D group. (f) ATP content of MSC in the 3D group and the 2D group. (g) The lactic acid content of MSC in the 3D group and the 2D group (*n* = 3). All data are shown as the mean ± SD, *p < 0.05; **p < 0.01; ***p < 0.001. MSC, mesenchymal stem cell; PGM‐HA, porous gelatin microcarrier crosslinked with hyaluronic acid.

SEM image suggested that MSC has already seeded on PGM‐HA (Figure 2a), and MSC still expressed stem cell markers CD29, and CD90 (Figure 2b). After planting MSC‐PGM‐HA on the monolayer dishes, MSC migrating from PGM‐HA remained a shuttle‐like morphology (Figure 2c). The results of the cell cycle reflected that the proportion of the G1 phase of the 3D group was significantly higher than that of the 2D group, while the proportion of the G2 phase was significantly lower, and there was no significant difference in the S phase of the two groups (Figure 2d). Cell apoptosis results suggested that MSC in the 3D group (5.23 ± 0.46%) showed a significantly lower apoptosis rate compared to MSC in the 2D group (6.90 ± 0.37%) (Figure 2e). Further exploration of metabolism was performed to test whether the 3D culture method would influence cells' oxidative stress. The ATP generation of the 3D group was significantly higher than that of the 2D group (Figure 2f), while the lactic acid content of the 3D group was lower than that of the 2D group (Figure 2g).

### 3.3 MSC‐PP acquisition by 3D dynamic culture system

**FIGURE 3:**
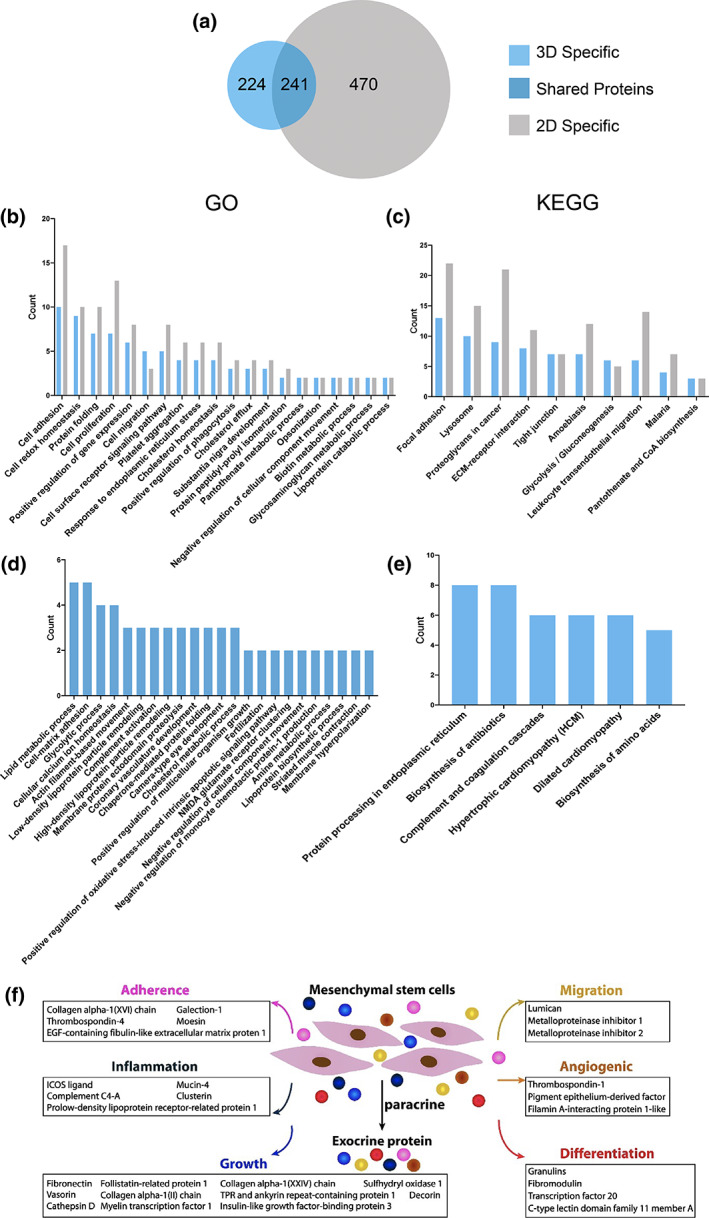
Component analysis of MSC‐PP. (a) Venn diagram showed the mass spectrometry result of MSC‐PP in the 3D group and the 2D group. (b) GO term enrichment analysis of proteins within the 3D group and the 2D group. (c) KEGG enrichment analysis of proteins within the 3D group and the 2D group. (d) GO term enrichment analysis of proteins specifically expressed in the 3D group. (e) KEGG enrichment analysis specifically expressed in the 3D group. (f) Protein function category of MSC‐PP in the 3D group. MSC‐PP, mesenchymal stem cell paracrine proteins.

Protein solution mass spectrometry analysis was conducted to identify specific protein species in the 3D group and the 2D group. The mass spectrometry analysis demonstrated that 465 and 711 protein species were identified in the 3D group and the 2D group, respectively. The two groups shared 241 protein species, approximately 52% of the total protein species in 3D (Figure 3a). In Figure 3b,d, GO analysis showed that the 3D group and the 2D group were respectively expressed in the common biological process, and a protein related to the biological process of cell‐matrix adhesion, glycolytic process, positive regulation of oxidative stress‐induced intrinsic apoptotic signaling pathway and negative regulation of monocyte chemotactic protein‐1 production were specifically expressed in the 3D group. In addition, the results of KEGG analysis showed that the common protein was expressed in the 3D group and the 2D group (Figure 3c,e), and the molecular functions of protein processing in the endoplasmic reticulum and biosynthesis of amino acids were specifically expressed in the 3D group, containing factors that promote cell proliferation and migration, enhance cell adhesion, and promote angiogenesis. Subsequent protein category revealed that MSC‐PP of the 3D group contains species that could promote cell proliferation and migration, and enhance cell adhesion, and angiogenesis (Figure 3f).

### 3.4 Effect of MSC‐PP on rabbit dermal fibroblasts and keratinocytes

**FIGURE 4:**
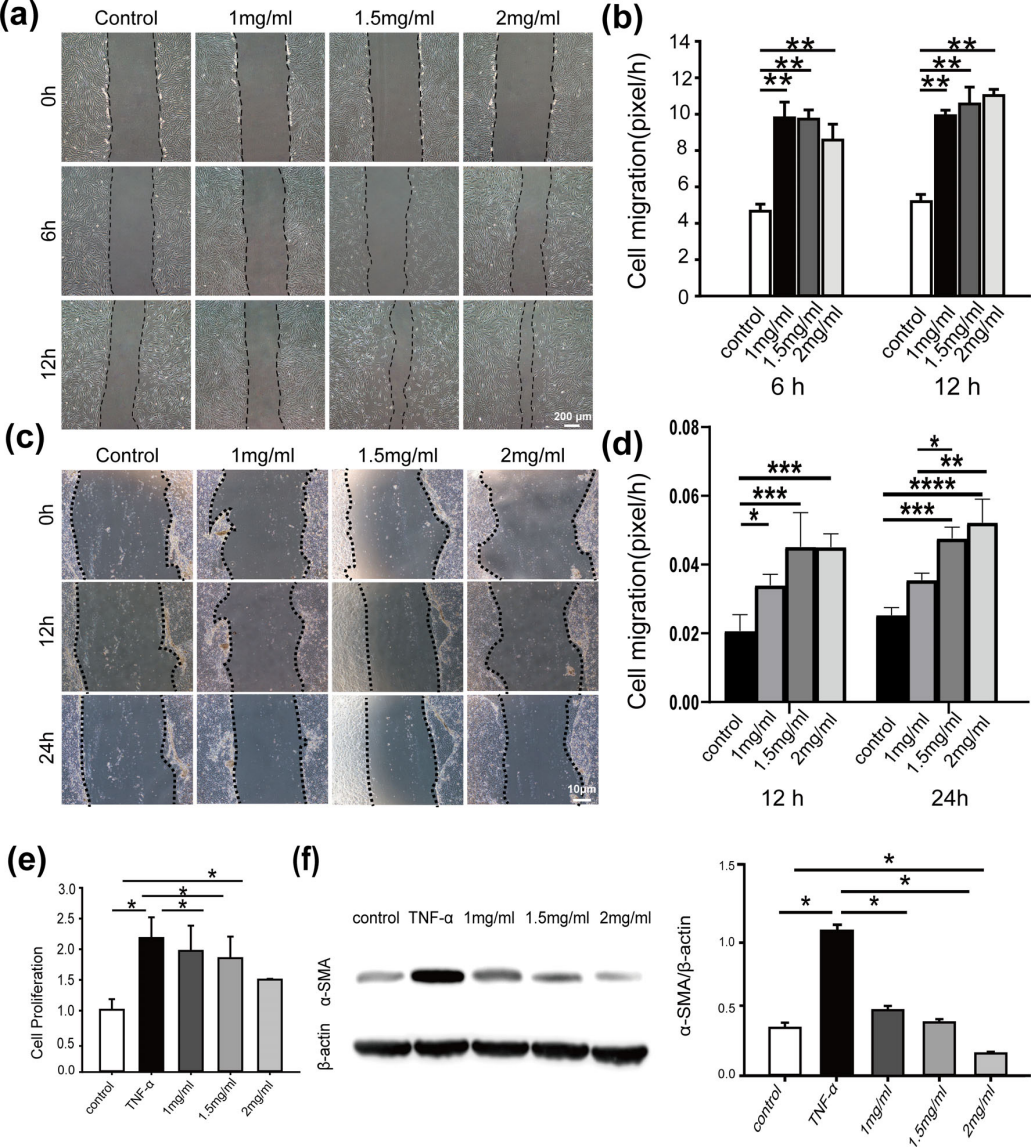
The effects of MSC‐PP on dermal fibroblasts and keratinocytes in vitro. **(**a) Migration ability of dermal fibroblasts treated with MSC‐PP for 0, 6 , and 12 h under serum‐free culture conditions. (b) The mobility of dermal fibroblasts at 6 and 12 h. (c) Migration ability of keratinocytes treated with MSC‐PP for 0, 12 and 24 h. (d) The mobility of keratinocytes at 12 and 24 h. (e) The effect of MSC‐PP on the proliferation efficiency of dermal fibroblasts in an inflammatory culture environment. (f) Western blotting results and corresponding quantification results of α‐SMA and β‐catenin (*n* = 3). All data are shown as the mean ± SD, *p < 0.05; **p < 0.01. MSC‐PP, mesenchymal stem cell paracrine proteins.

The effects of MSC‐PP on dermal fibroblast and keratinocytes migration ability were assayed. According to the results, the migration rate of rabbit dermal fibroblasts in the MSC‐PP treatment groups at 6 and 12 h were significantly higher than that of the control group, respectively (Figure 4a,b). In addition, the migration rate of keratinocytes of the MSC‐PP treatment groups were also significantly higher than that of the control group at 12 and 24 h (Figure 4c,d). The effect of MSC‐PP on the dermal fibroblast proliferation under TNF‐α induced inflammatory microenvironment was also observed. Significantly lower proliferation rates of dermal fibroblasts in the MSC‐PP groups were observed following the treatment of TNF‐α within 24 h, and the fibroblast proliferation rate decreased along with adding the concentration of MSC‐PP (Figure 4e). Moreover, compared to the control group, dermal fibroblast showed a significant increase of α‐SMA expression level in the TNF‐α group, while the treatments of MSC‐PP showed significantly lower expression levels of α‐SMA than the TNF‐α group (Figure 4f).

### 3.5 Characteristics of PEG temperature‐sensitive hydrogel

**FIGURE 5:**
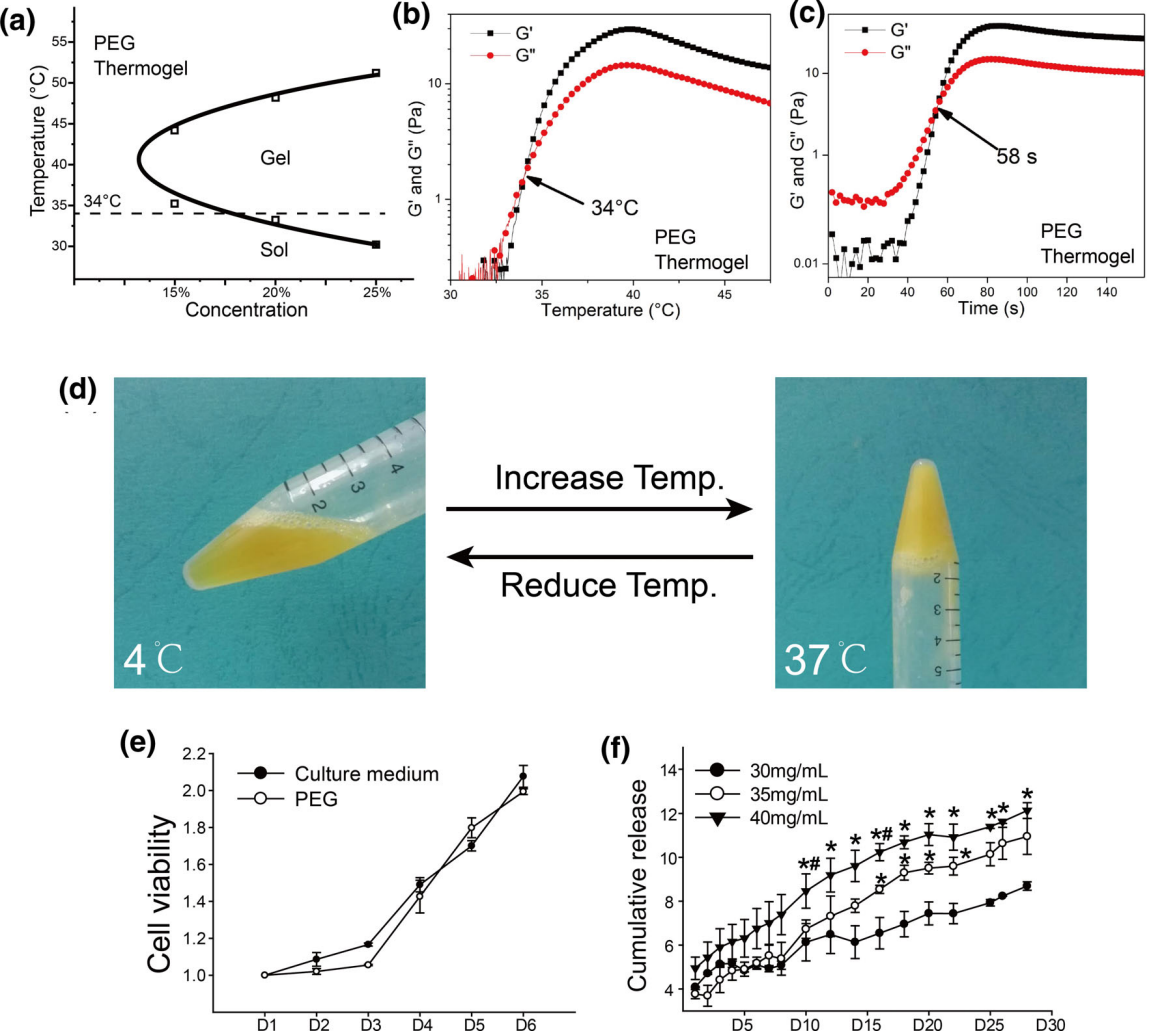
Characterization of PEG thermosensitive hydrogel. (a) Solution‐gel phase transition of PEG thermogel. (b) Rheological detection of PEG thermogel. (c) Rheological detection of PEG thermogel with time. (d) Different physical forms of PEG thermogel at 4 and 37°C. (e) PEG thermogel cell toxicology test over 5 days. (f) The sustained release efficiency of MSC‐PP in PEG thermogel at 28 days (*n* = 3). All data are shown as the mean ± SD, *p < 0.05 versus 30 mg/mL; ^**#**^ *p* < 0.05 versus 35 mg/mL. PEG, polyethylene glycol; MSC‐PP, mesenchymal stem cell paracrine proteins.

Micelle aqueous transitions of PEG hydrogel in 15%, 20%, and 25% are shown sol‐to‐gel transitions (lower transition) and gel‐to‐sol transitions (upper transition) as temperature increases, and the gel window of the PEG hydrogel micelle aqueous solution contained 37°C (Figure 5a,d). This trend in sol–gel transition has been reexamined by the rheological measurement of copolymer aqueous solutions under temperature sweep (Figure 5b) and time sweep (Figure 5c). The shear moduli of PEG aqueous solutions with temperature were presented in Figure 5b, which appears the gelation temperature was 32.5°C. And the shear moduli of PCT‐PEG‐PCT aqueous solutions with time at 37°C were presented in Figure 5c, which appeared the gelation time was 45 s at body temperature. The cell toxicity experiment revealed no significant difference between the normal culture medium and the PEG immersion culture medium within 5 days (Figure 5e), indicating that the PCT‐PEG‐PCT had no cytotoxicity and did not interfere with cell proliferation. The release of MSC‐PP in PEG hydrogel was stable, without significant fluctuations, and the release of MSC‐PP in the 40 mg/mL group was maintained at 0.373 ± 0.153 mg/day (Figure 5f).

### 3.6 MSC‐PP + PEG improved wound contraction and reduced scarring

**FIGURE 6:**
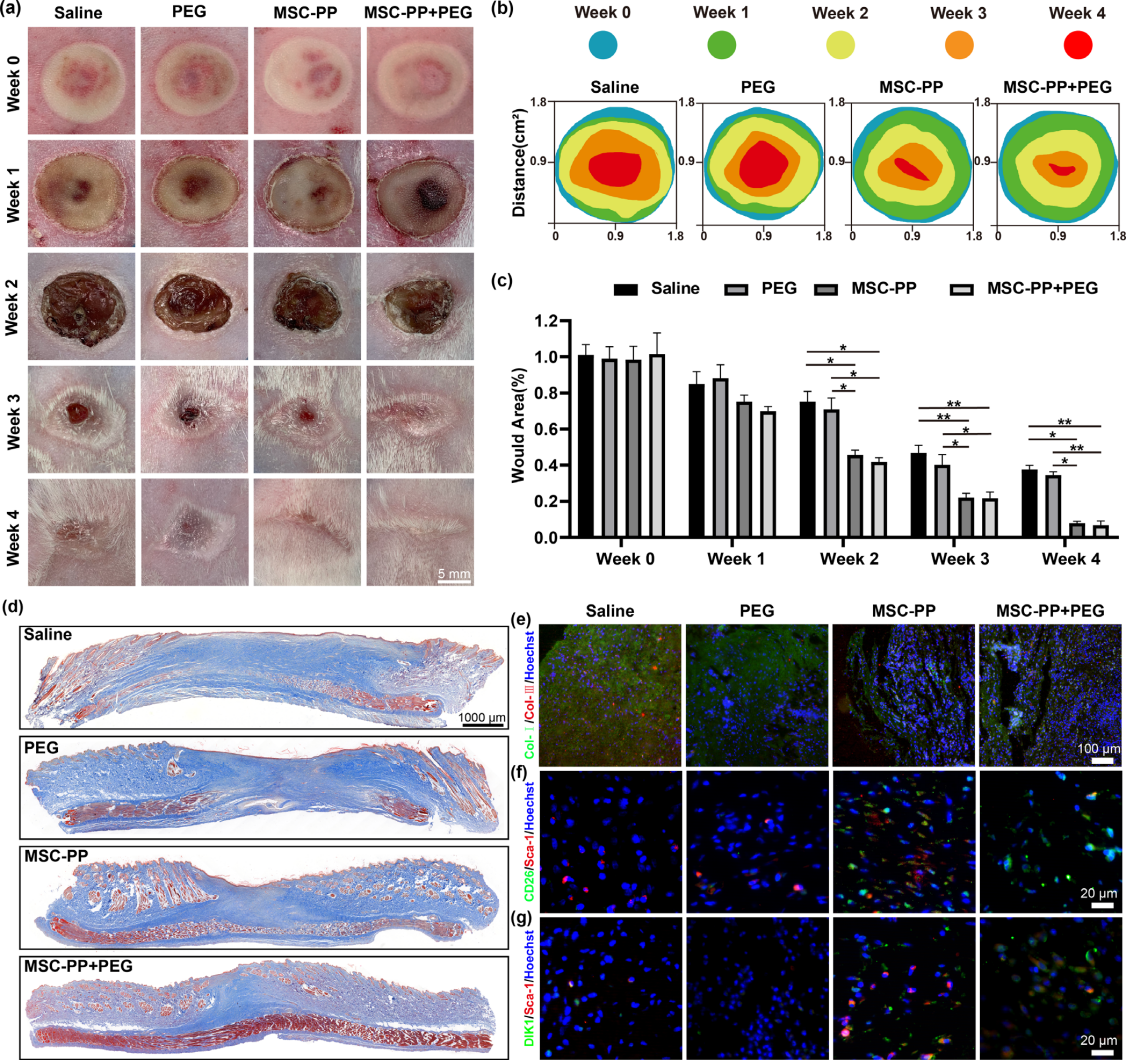
MSC‐PP + PEG facilitated wound contraction and inhibited scar formation in the rabbit third‐degree burn models. (a) Representative photographs of wounds treated with saline, PEG, MSC‐PP, and MSC‐PP + PEG at week 0, 1, 2, 3, and 4. (b) Color images of the patterns of the wounds during the 4 weeks of treatment. (c) Percentages of treated wound areas over 4 weeks. (d) Masson staining results of four groups at week 4. (e) Immunofluorescence staining results of type I and type III collagen at week 4. (f) Immunofluorescence staining results of CD26+/Sca‐1^+^ cells at week 4. (g) Immunofluorescence staining results of Dlk‐1+/Sca‐1^+^ cells at week 4 (*n* ≥ 3). All data are shown as the mean ± SD, *p < 0.05; **p < 0.01. PEG, polyethylene glycol; MSC‐PP, mesenchymal stem cell paracrine proteins.

To evaluate the effect of MSC‐PP + PEG on wound healing, wound contraction was observed and calculated every week (Figure 6a–c). Following scalding, the wounds of all groups were festering at week 1 and formed scabs at week 2, and significant differences in wound contractions were observed from week 2 to week 4. The MSC‐PP + PEG group showed the most effective wound contraction among the four groups. The scabs in the MSC‐PP + PEG group peeled off and completed re‐epithelialization in week 3, with a minimum scar size in week 4, while the saline group and the PEG group still had obvious scars in week 4 (Figure 6a). Furthermore, the wound area in the MSC‐PP group and the MSC‐PP + PEG group were significantly smaller than those in the saline group and the PEG group from week 2 to week 4, but no significant difference was observed between the MSC‐PP group and the MSC‐PP + PEG group (Figure 6b,c). Consistency to the wound area calculation results, collagen deposition in the MSC‐PP + PEG group significantly decreased and showed the smallest scar areas compared to the other three groups (Figure 6d), and the ratio of collagen I/III expression levels of the MSC‐PP group and the MSC‐PP + PEG group were significantly lower than those of the saline group and the PEG group, respectively (Figure 6e; Figure S1). Moreover, CD26^+^/Sca‐1^+^ cells and Dlk‐1^+^/Sca‐1^+^ cells were important for inhibiting scar formation, and they were barely seen in the saline group and PEG group, while the highest amount of CD26^+^/Sca‐1^+^ cells and Dlk‐1^+^/Sca‐1^+^ cells were observed in the MSC‐PP + PEG group (Figure 6f,g).

### 3.7 MSC‐PP + PEG reduced inflammation, accelerated wound re‐epithelialization, promoted skin appendages recovery and angiogenesis

**FIGURE 7:**
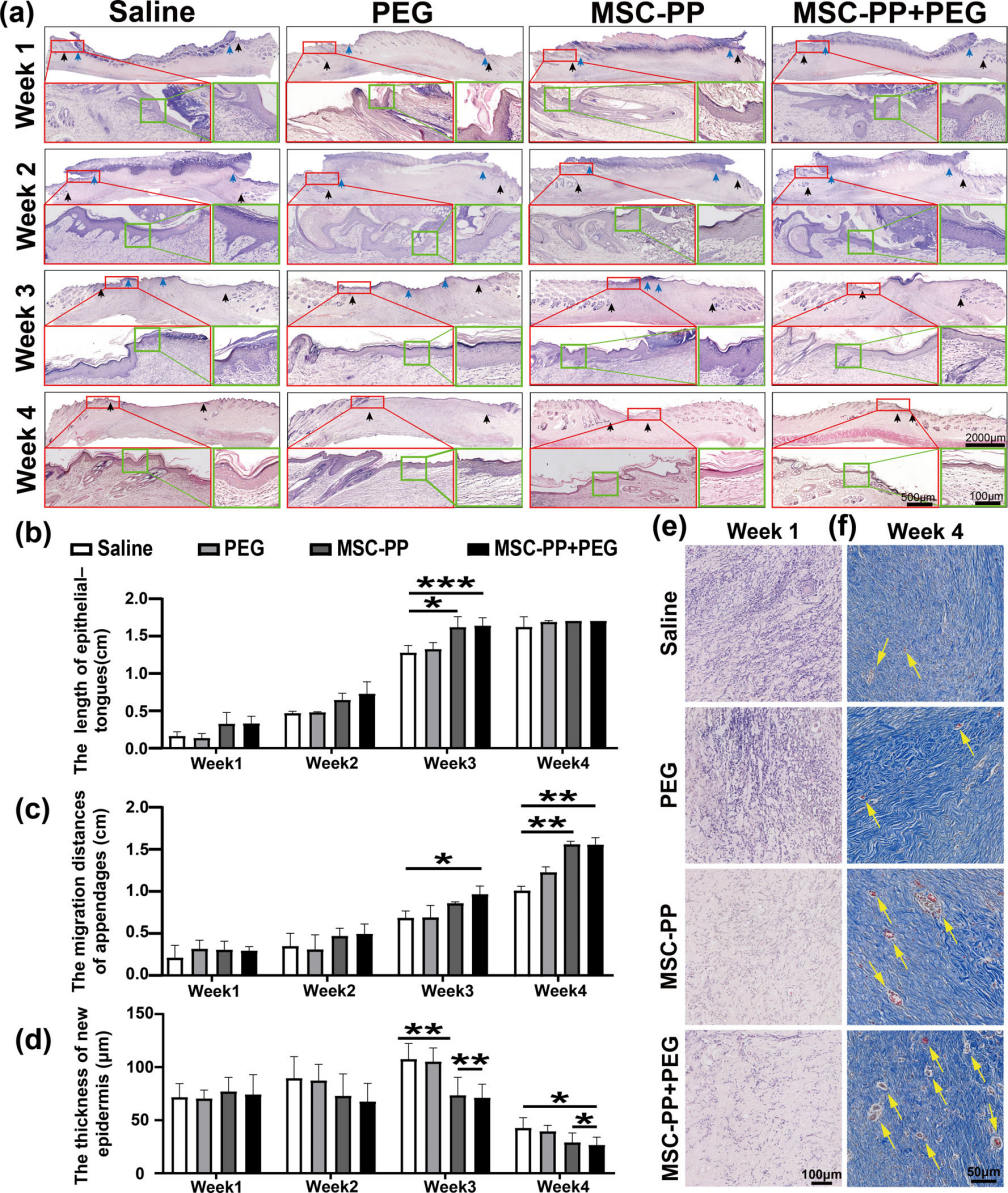
Histological analysis evidence of MSC‐PP + PEG improved deep burn wound healing and skin appendage regeneration. (a) H&E results of the deep burn wound tissues every week. Red boxes show the formation of epidermis and hair follicles during wound re‐epithelialization, green boxes indicate the thickness of the new epidermis, blue arrows indicate the front edges of the migrated epithelial tongues, and black arrows indicate the front edges of newly formed skin appendages. The scale bars in the graphs are 2000, 500, and 100 μm, respectively. (b) The calculated migration length of the epidermal tongues each week in the different treatment groups. (c) Migration distances of regenerated skin appendages each week in the different treatment groups. (d) Measurement of the new epidermis thickness. (e) Representative results of inflammatory cells infiltration at week 1. (f) Angiogenesis within the wound area at week 4, and the yellow arrows indicate the vessel‐like structures (*n* = 3). All data are shown as the mean ± SD, *p < 0.05; **p < 0.01; ***p < 0.001. PEG, polyethylene glycol; MSC‐PP, mesenchymal stem cell paracrine proteins.

Skin samples from each group at each week point were evaluated by H&E staining to histologically assess the wound healing. Compared to the saline group, the significant increase in the migration length of epithelial tongues at week 3 in the MSC‐PP group and the MSC‐PP + PEG group (Figure 7a,b). Moreover, new inner and outer root sheaths structures were obviously seen in the newly formed hair follicles in the MSC‐PP + PEG at week 3, and the new skin appendages had moved to the center of the wound in week 4, but there was no discernible migration and recovery in the saline and the PEG groups. In addition, a significant difference in the migration of new skin appendages between the MSC‐PP group and the saline group was observed in week 4 (Figure 7a,c). Increases of epidermal thickness were observed in all groups following 4‐week repairing, and the quantification results of the epidermal thickness showed that the MSC‐PP group and the MSC‐PP + PEG group significantly reduced epidermal thickness compared to the saline group, and the MSC‐PP + PEG group showed the lowest epidermal thickness compared to the other three groups, which was close to the thickness of healthy skin (Figure 7a,d). Besides, inflammatory cells infiltration could be observed around the wound area at week 1, while MSC‐PP group and MSC‐PP + PEG group exhibited relatively less inflammatory cells around the wound area compared to the saline group (Figure 7e). Vessel‐like structures were clearly observed within MSC‐PP and MSC‐PP + PEG treatment at week 4 (Figure 7f), and related quantitative results also showed that MSC‐PP + PEG group had the most vessel‐like tissues (Figure S2). These results demonstrated that the MSC‐PP and MSC‐PP + PEG groups could improve re‐epithelialization, facilitate skin appendage recovery, reduce early inflammation, and promote angiogenesis at the wound area.

### 3.8 The role of β‐Catenin, KRT5, and LGR6 regulatory differentiation on skin appendages

**FIGURE 8:**
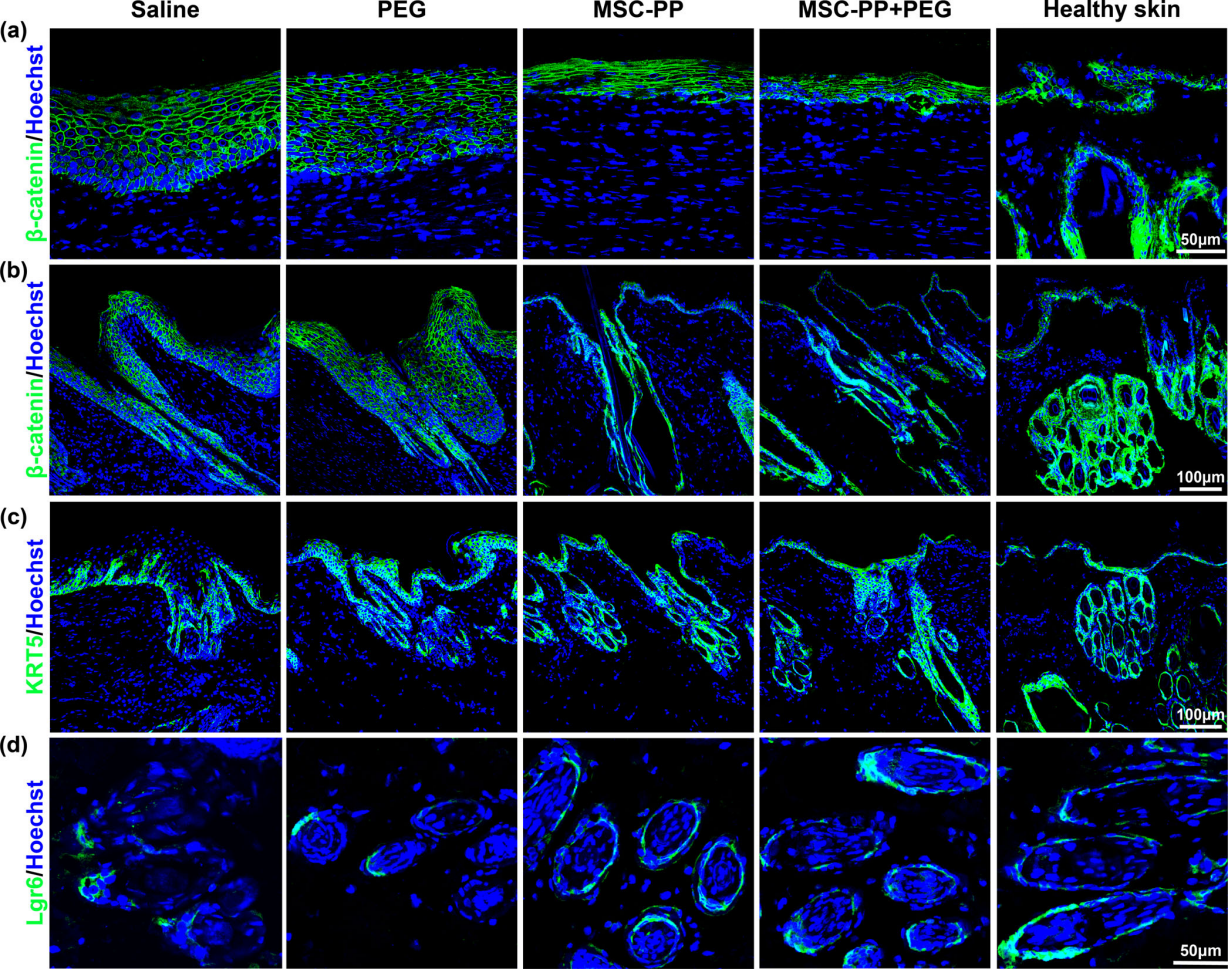
MSC‐PP + PEG induced multiple cells to be involved in wound repair. (a) Immunofluorescence expression of β‐Catenin in the newly formed epidermis and hair follicles at week 4 in the different treatment groups. (b–d) The expression of KTR5^+^cells and LGR6^+^cells in nascent hair follicles in the different treatment groups at week 4 (*n* = 3). PEG, polyethylene glycol; MSC‐PP, mesenchymal stem cell paracrine proteins.

As an important regulator for hair follicle generation, the expressions of β‐Catenin in the recovered epidermis 4 weeks following treatments were observed. In the saline group and the PEG group, positive β‐Catenin signals were observed in the full‐thickness of the thickened epidermis, and similar expression patterns were observed in the downward grew epidermis, primary structures of the neogenesis hair follicles. For the MSC‐PP group and the MSC‐PP + PEG group, positive β‐Catenin signals were also observed in the epidermis, and strong β‐Catenin signals could be widely observed in the neogenesis hair follicles with mature structures, which were similar to the expression patterns in the healthy skin epidermis and hair follicles (Figure 8a,b). Subsequently, KRT5^+^ cells, epidermal stem cells associated with epidermal and hair follicle neogenesis, were examined. KRT5^+^ cells were expressed in the thickened epidermis of both the saline group and the PEG group. However, due to the thinness of the nascent epidermis in the MSC‐PP and the MSC‐PP + PEG group, KRT5^+^ cells were not only expressed in the epidermis but were more involved in the structure formation of the nascent hair follicles (Figure 8c). In addition, LGR6^+^ cells were observed in all the treatment groups (Figure 8d).

## 4 DISCUSSION

To obtain high‐quality MSC‐PP, a 3D dynamic culture system was established with modified PGM by crosslinking HA and the specific function of MSC‐PP was analyzed. The PEG thermogel was modified by introducing the cyclic ether side group to reduce the crystallinity and gelation behavior as a carrier to deliver MSC‐PP. The mixture of MSC‐PP and PEG thermogel was transplanted into the third‐degree burn rabbit models. Compared to the other three groups, the MSC‐PP + PEG group significantly improved skin function.

Porous microcarriers could provide large surface area for cell attachments and proliferation, and establishing large‐scale MSCs culture system with PGM could easily acquire cells and secreted products.^22^ The coating of cell microcarriers could enable surface cell adherence to be strong enough to support cell growth under shear stress conditions in a bioreactor.^23^ In this study, HA could generate connection and action through chemical bonds with collagen, and we successfully crosslinked HA on PGM, and the porous collagen ultrastructure was well preserved. It was reported that the water absorption rate would influence the chitosan swelling ability and structure, increasing the adhesion of hepatocytes accordingly.^24^ The absorption rate of PGM‐HA was significantly enhanced, which showed stronger hydrophilicity, indicating that PGM‐HA might improve cell adhesion ability. Moreover, PGM‐HA showed no biotoxicity. Thus, the PGM‐HA was effectively prepared and could be used as microcarriers in a 3D‐dynamic culture system. Previous studies about using a 3D culture system to expand cells showed that the cell expansion ratio was between 2.60‐fold and 21.00‐fold in 10 days.^25^, ^26^ In our study, we achieved expanding cells 11‐fold in 9 days, at the same time, the culture medium components were also stable during the culture process. Accordingly, these results showed that we have already prepared a 3D dynamic culture system.

The dynamic culture system could provide fluid shear force, achieve better nutrient exchange and promote cell proliferation and maintain stem cell bioactivates.^27^ In the present study, MSC could grow on PGM‐HA and maintain its stem cell properties with enough nutrition supply generated by a 3D dynamic culture system. Despite stem cell properties, optimizing stem cell metabolism was an important issue. Due to the increasing attach surface area and enough nutrition supply from the dynamic culture system, cells of the 3D group showed more G1 phase proportion than the 2D group, indicating that cells cultured in the 3D dynamic system were more active in material metabolism and rapidly synthesize RNA and protein than 2D culture dishes.^28^, ^29^ Furthermore, the MSC apoptosis rate in the 2D group reached 6.90%, while only a 5.23% cell apoptosis rate was observed in the 3D group. Under the circumstances, oxygen and nutrition deficiencies in the 2D group were likely to be the major reason for the higher apoptosis, because the culture medium flowed continuously through MSC‐PGM‐HA in the dynamic culture system, providing enough nutrition and carrying out the metabolic waste.^30^ As a result, with enough nutrition supply, MSC in the 3D group could grow faster having a higher ATP production, and a lower lactic acid content.

MSC‐PP was influenced by the bioactivity of MSC culturing in the 3D group and the 2D group. Although MSC could proliferate in the 2D culture system, the 2D culture system is not enough to simulate the three‐dimensional microenvironment in vivo, affecting the apoptosis and metabolism of MSC.^31^, ^32^ Through mass spectrometry analysis, shared proteins account for 52% of the total protein species including laminin, growth factors, collagen, and fibronectin, which has already been reported before.^8^, ^33^ We found that there were 470 more protein types in the 2D culture group than in the 3D culture group, but almost all of these 2D‐specific proteins are proteins caused by apoptosis or stress metabolism in the 2D culture process, which play a negative regulatory role in biological processes such as cell adhesion and cell proliferation (such as RHOB, RAC1, etc.), and even cause excessive inflammatory response (such as CD47, CCL2, etc.).^34^, ^35^ Although there are only 465 protein species in the 3D group, among which these proteins can specifically promote the mediation of cell attachment and migration (such as LAMA1, POSTN, TLN1, etc.), promote cell proliferation (such as PRKDC, ASCC3, MYH10, PDK1, etc.), regulate oxidative stress‐induced intrinsic apoptosis signaling pathways (such as SFPQ, PARK7), and so forth.^36^, ^37^, ^38^, ^39^, ^40^ Moreover, proteins related to cell metabolism and skin regeneration and repair, such as fibromodulin and IGF, were also found in the 3D group by MS analysis.^41^, ^42^ This may be related to the 3D environment allowing greater cell‐to‐cell contact, leading to increased intercellular signaling, promoting developmental processes, and allowing cells to differentiate into more complex structures, bringing cell viability closer to their physiological state, favoring the exertion of their paracrine effects. By providing an in vitro physiological culture environment for large‐scale MSC production, favorable proteins which was beneficial for wound healing could be efficiently obtained. Therefore we used MSC‐PP obtained from the 3D culture group in the subsequent experiments and treatments.

The purified MSC‐PP contained a variety of cytokines that can play a synergistic role in promoting cell migration, so the addition of MSC‐PP could effectively promote dermal fibroblasts and keratinocytes migration. The effect of MSC‐PP on the dermal fibroblasts under a TNF‐α inflammatory environment was observed, and the results illustrated that the dermal fibroblasts overproliferated two times compared to the normal group under TNF‐α. At the same time, the proliferative effect of TNF‐α on the dermal fibroblasts decreased with the increase of MSC‐PP concentration. It was reported that TNF‐α could regulate proliferation, survival, and apoptosis via the MAPK pathway, while MSC‐PP may prevent TNF‐α‐induced phosphorylation of p38.^43^ The western blot further showed that MSC‐PP could reduce the α‐SMA expression of dermal fibroblasts. α‐SMA is mainly expressed by myofibroblasts in dermal tissue. The above results suggested that MSC‐PP might rapidly achieve wound re‐epithelialization and reduce scars by promoting keratinocytes migration, inhibiting dermal fibroblasts over proliferation and conversion to myofibroblast cells.

To reduce the loss of MSC‐PP in the wound area, we chose hydrogel to deliver drugs because of its appropriate mechanical properties, injectable capacity, and excellent cell biocompatibility, and achieving sustained release effect of the drug and reducing the number of repeated dosing remains significant challenges.^44^, ^45^, ^46^, ^47^ In the present study, we successfully prepared the PEG thermosensitive hydrogel that enabled sustained release of MSC‐PP within 4 weeks. PEG thermogel was modified from the existing injectable PEG hydrogel, which can reduce the polymer's crystallinity, lower the gelation temperature, shorten the gelation time, and reduce the gel modulus after gelation. The PEG thermogel had the thermosensitive characteristic to avoid the problem to form spontaneous crystalline gel before injection.^48^ The results of co‐culture with dermal fibroblasts have further confirmed it. The MSC‐PP was added to the hydrogel to construct a sustained‐release system. The sustained‐release system could stably release the protein within 4 weeks, indicating that the MSC‐PP thermosensitive hydrogel sustained‐release system was successfully established.

Subsequently, the MSC‐PP + PEG was injected into the rabbit third‐degree burn model to evaluate the sustained‐release effect. There are three main sequential but partially overlapping phases of wound healing: inflammation, epithelialization, and tissue remodeling.^49^ The persistent inflammatory response, tissue ischemia, and hypoxia are critical factors affecting wound repair and may lead to excessive scar formation. Timely regulation of the inflammatory response could make the wound quickly transition from inflammatory stage to the recovery stage. Also, improving angiogenesis during the wound repair process could recover nutrients supply and transport metabolic wastes within the wound area, which is essential for wound healing.^50^, ^51^ Studies have shown that paracrine proteins of stem cells can accelerate wound healing by immunomodulation and promoting angiogenesis.^52^ Histological analysis of the present study also showed that MSC‐PP + PEG treatment reduced inflammatory cells infiltration, and the most blood vessels were observed in the MSC‐PP + PEG group. To sum up, MSC‐PP and MSC‐PP + PEG could reduce inflammation and promote angiogenesis during the wound repair process of third‐degree burn model.

Re‐epithelialization was also essential for wound repair and recovery of skin integrity.^53^, ^54^, ^55^ It was reported that stem cell paracrine proteins therapy could accelerate wound re‐epithelization and wound healing by promoting cell proliferation and tissue remodeling.^8^, ^56^ In this study, the MSC‐PP + PEG group had the smallest wound area and the fastest re‐epithelialization rate during wound repair. Researchers found that co‐transplantation of MSCs with simvastatin in the rat burn model increased the thickness of the nascent epidermis after 2 weeks.^57^ However, it has been reported that the faster the rate of re‐epithelialization, the thinner the epidermis, leading to reduce of scar formation.^58^ In our study, the thickness of the nascent epidermis was observed to increase and then decrease. By the fourth week, epidermal thickness was significantly reduced in the MSC‐PP and MSC‐PP + PEG groups compared to the saline group. Thus, with the fastest wound contraction and re‐epithelialization, and the thinnest epidermal thickness following third‐degree burn, the MSC‐PP + PEG group could provide a good basis for further collagen rearrangement and scar reduction.

During the tissue remodeling phase of the wound, an imbalance in the collagen ratios would cause severe scarring, leading to the loss of skin elasticity and physiological function after wound repair. Late delayed re‐epithelialization and thicker epidermis postponed collagen remodeling, resulting in the accumulation of irregular collagen fibers, which would increase scar formation.^58^ Consistently, MSC‐PP + PEG group showed rapid re‐epithelialization and significant inhibition of scar formation in this study. In addition, it was reported that fibroblasts in the skin were mainly divided into two subspecies that might determine the recovery of wounds, which were reticular fibroblasts (Dlk‐1^+^/Sca‐1^+^ or Dlk‐1^−^/Sca‐1^+^) and papillary dermal fibroblasts (CD26^+^/Sca‐1^+^).^59^, ^60^, ^61^ They have a strong activity and contribute to all skin mesenchyme compartments. The results showed that Dlk‐1^+^/Sca‐1^+^ cells and CD26^+^/Sca‐1^+^ cells could be observed in the MSC‐PP + PEG group, while there was almost no expression of Dlk‐1^+^/Sca‐1^+^ cells and CD26^+^/Sca‐1^+^ cells in the healing sites of the saline group and the PEG group, indicating that the healing sites were mainly filled by myofibroblasts, which constituted skin tissue with a certain thickness, but did not have normal functional cortical structure. The above results indicated that the MSC‐PP + PEG sustained‐release system could promote tissue remodeling and significantly reduce the scar area.

Besides scar inhibition, improving skin appendage regeneration was another important issue for deep burn skin recovery. The research reported that skin damage would activate the hair follicle's embryonic regeneration program.^62^, ^63^ In the nascent epidermal, epidermal basal stem cells would gradually thicken and grow downward to form the placode, which was the hallmark event of hair follicle embryonic regeneration.^64^ Notably, the Wnt signaling system is required for regulating cell proliferation and differentiation, as well as regeneration and repair following injury. β‐Catenin was the canonical effector molecule in the Wnt signaling pathway and a key regulator for controlling the cell fate of hair follicle generation.^65^, ^66^, ^67^ In addition, the interaction of various involved cells and pathways would also affect the hair follicles regeneration process, such as the activation KRT5^+^ cells and LGR6^+^ cells.^7^, ^68^, ^69^ These regulatory proteins were observed in both the nascent epidermis and hair follicles in the four treatment groups. In the MSC‐PP and MSC‐PP + PEG groups, the expression of β‐catenin and KRT5 was obviously seen in the regenerated hair follicles in the wound area, and more functional hair follicles were observed in these two groups. But in the saline group and the PEG group, the over‐thickened epidermis may not be advantageous for the occurrence of the hallmark event of hair follicles formation. This suggested that MSC‐PP and MSC‐PP + PEG may promote the differentiation of basal stem cells to hair keratinocytes by activating the Wnt signaling pathway and regulating the expression of the target gene β‐catenin. The specific molecular mechanism will need to be verified in more subsequent experiments. Taken together, it was reasonable to conclude that MSC‐PP + PEG could promote the healthy and mature skin appendages regeneration by regulating the critical regulators associated with the hair follicles, which could better restore the regulatory, secretory, and excretory function of the skin, and this also showed great potential for the treatment of hair regeneration.

In general, MSC‐PP from 3D dynamic culture systems had a variety of proteins that promoted cell growth and regulated signaling pathways. Combined with PEG thermogel, MSC‐PP could release in the wound stably and sustainably, which could greatly lessen drug administrations during treatment. Moreover, the MSC‐PP thermosensitive hydrogel system significantly improved deep burn skin wound healing, efficiently restored epidermal barrier function, reduced scar formation, and facilitated the regeneration of skin appendages, these therapeutic effects on small deep burns of MSC‐PP + PEG may offer good ideas and methods for the treatment of large deep burns.

## 5 CONCLUSIONS

In summary, the 3D dynamic culture system could obtain MSC‐PP with high quality, and PEG thermogel was used as the injection carrier of MSC‐PP, and the conformability on skin tissues and the sustained‐release effect of PEG thermogel offered MSC‐PP a sustained and stable treatment on a skin wound. The study emphasized the importance of the continued effect of MSC‐PP on wound regeneration, which can regulate the composition and proportion of the extracellular matrix, improve wound healing rate, inhibit scar formation, and facilitate skin appendage regeneration.

## AUTHOR CONTRIBUTIONS

**Yingwei Wang:** Conceptualization (lead); data curation (lead); formal analysis (lead); writing – original draft (lead). **Jiaxin Wu:** Conceptualization (lead); data curation (lead); formal analysis (lead); investigation (lead); writing – original draft (lead). **Jiamin Chen:** Data curation (equal); software (equal); writing – original draft (equal); writing – review and editing (equal). **Cheng Lu:** Conceptualization (equal); data curation (equal); software (equal); writing – original draft (equal). **Jinchao Liang:** Investigation (equal); methodology (equal); writing – original draft (equal). **Yingyi Shan:** Methodology (equal); project administration (equal); software (equal). **Jie Liu:** Investigation (equal); methodology (equal); writing – original draft (equal). **Qi Li:** Conceptualization (equal); formal analysis (equal); methodology (equal); software (equal). **Liang Miao:** Formal analysis (equal); writing – original draft (supporting). **Mu He:** Formal analysis (equal); writing – original draft (supporting). **Xiaoying Wang:** Data curation (supporting); methodology (supporting); writing – original draft (supporting). **Jianhua Zhang:** Conceptualization (supporting); funding acquisition (equal); resources (lead); writing – review and editing (supporting). **Zheng Wu:** Conceptualization (lead); funding acquisition (lead); methodology (supporting); supervision (lead); writing – review and editing (supporting).

## CONFLICT OF INTEREST STATEMENT

All authors involved in this article declare that there are no conflicts of interest regarding the publication of this paper.

### PEER REVIEW

The peer review history for this article is available at https://www.webofscience.com/api/gateway/wos/peer-review/10.1002/btm2.10569.

## Supporting information


**FIGURE S1.** Semi‐quantitative results of collagen immunofluorescence staining. Fluorescence intensity of collagen immunofluorescence staining in saline, PEG, MSC‐PP, and MSCP‐PP + PEG groups.(*n* = 3). All data are shown as the mean ± SD, *p < 0.05; **p < 0.01.
**FIGURE S2.** Quantitative results of the number of vessel‐like tissues.(*n* = 3).All data are shown as the mean ± SD, *p < 0.05; **p < 0.01; ***p < 0.001.

## Data Availability

The datasets used and/or analyzed during the current study are available from the corresponding author on reasonable request.
