# Supplementary material for: Mesenchymal stem cells paracrine proteins from three‐dimensional dynamic culture system promoted wound healing in third‐degree burn models
Source: Bioeng Transl Med. 2023 Jul 2;8(6):e10569. doi: 10.1002/btm2.10569 (PMC10658564; doi:10.1002/btm2.10569)
Supplement: Supplementary file 1 — FIGURE S1. Semi‐quantitative results of collagen immunofluorescence staining. Fluorescence intensity of collagen immunofluorescence staining in saline, PEG, MSC‐PP, and MSCP‐PP + PEG groups.(n = 3). All data are shown as the mean ± SD, *p < 0.05; **p < 0.01. FIGURE S2. Quantitative results of the number of vessel‐like tissues.(n = 3).All data are shown as the mean ± SD, *p < 0.05; **p < 0.01; ***p < 0.001. [file BTM2-8-e10569-s001.docx]

**Supplementary Information**

**MSCs Paracrine Proteins from 3D Dynamic Culture System Promoted Wound Healing in Third-degree Burn Models**

Yingwei Wang^a,b#^, Jiaxin Wu^b#^, ,Jiamin Chen^b^, Cheng Lu^b^, Jinchao Liang^b^, Yingyi Shan^b^, Jie Liu^b^, Qi Li^b^, Liang Miao^e^, Mu He^e^, Xiaoying Wang^c^, Jianhua Zhang^d*^, Zheng Wu^b*^

a Department of Ophthalmology, The First Affiliated Hospital of Jinan University, Guangzhou, 510630, China

b Key Laboratory for Regenerative Medicine, Ministry of Education, Department of Developmental and Regenerative Biology, Jinan University, Guangzhou, 510632, China

c Department of Biomedical Engineering, Jinan University, Guangzhou, 510632, China

d Special Wards, The First Affiliated Hospital of Jinan University, Guangzhou, 510630, China

e Burn plastic surgery, Longgang Central Hospital, Shenzhen, 518000,China

**# These authors contributed equally to this research.**

**
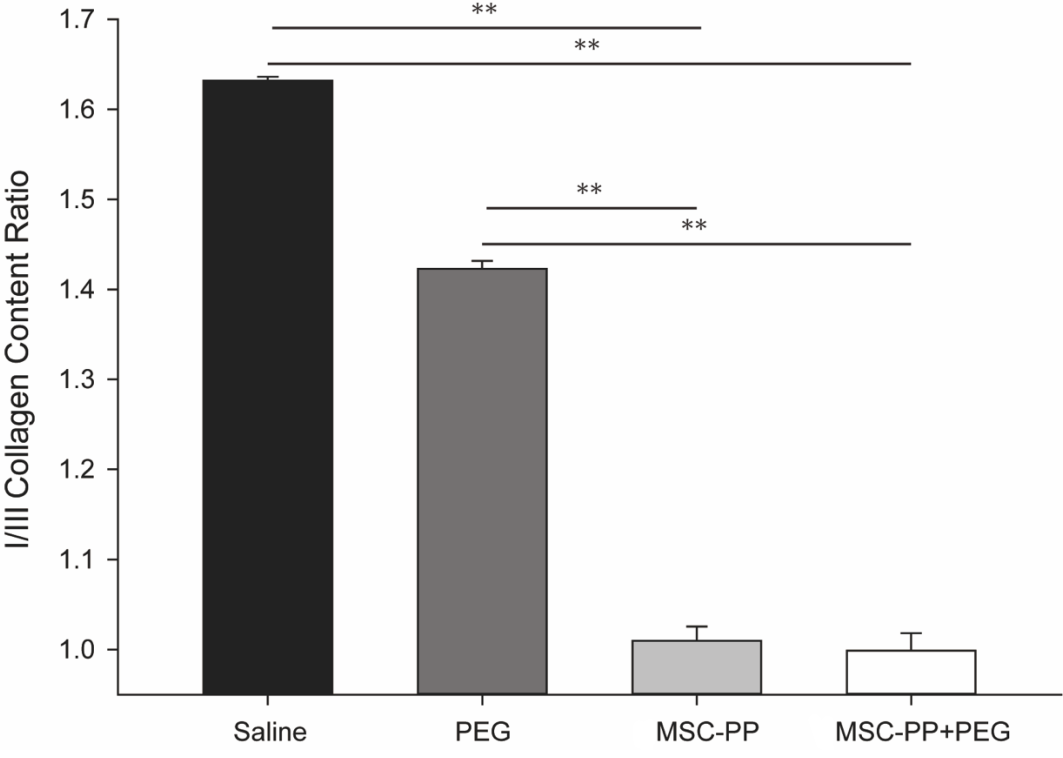
**

**FIGURE S1. Semi-quantitative results of collagen immunofluorescence staining.** Fluorescence intensity of collagen immunofluorescence staining in saline, PEG, MSC-PP and MSCP-PP+PEG groups.(*n* = 3). All data are shown as the mean ± SD, **P*< 0.05, ***P*<0.01.


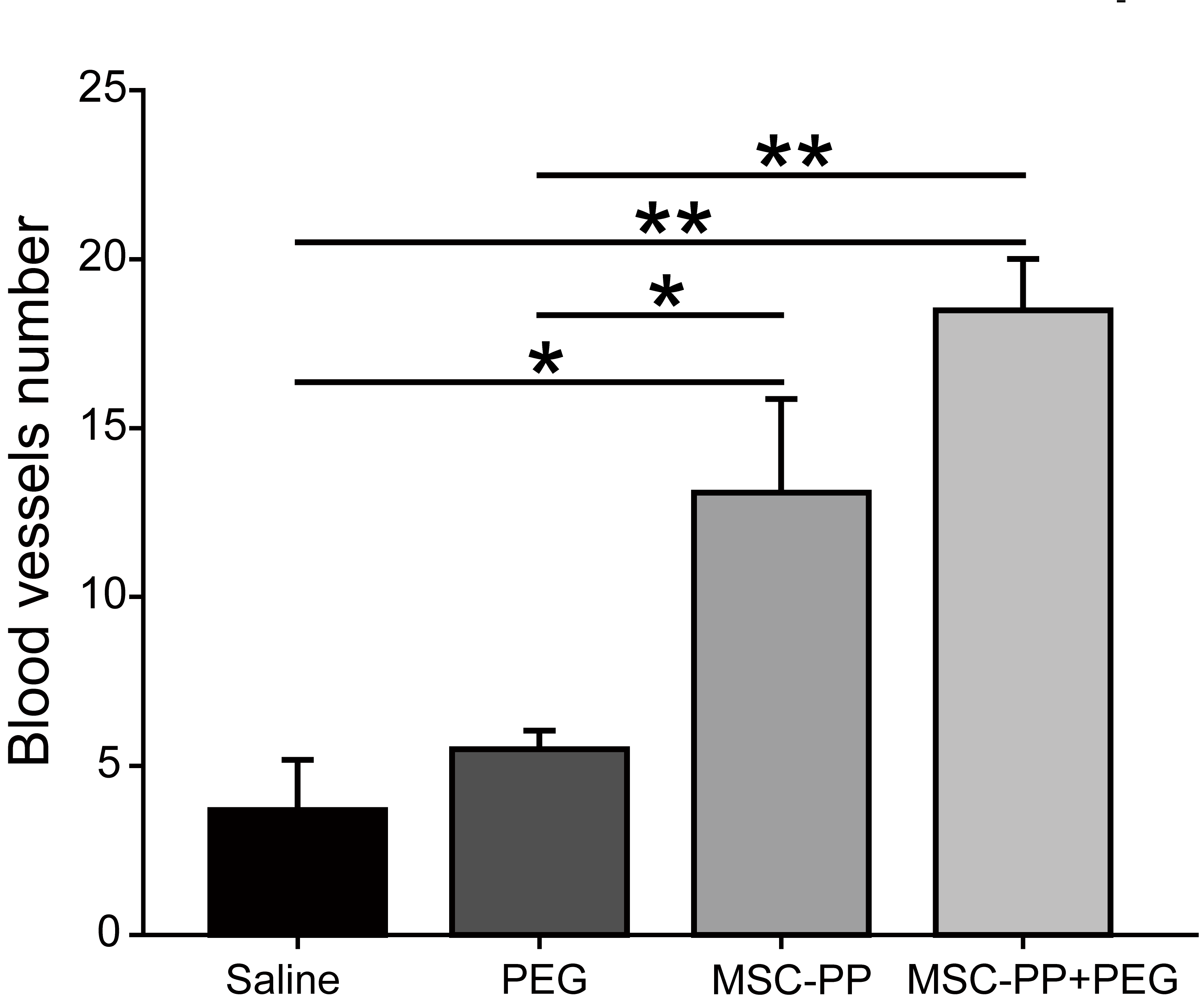


**FIGURE S2.** Quantitative results of the number of vessel-like tissues.(*n* = 3).All data are shown as the mean ± SD, **P*< 0.05, ***P*<0.01, ****P*<0.001.
